# A high-resolution global annual city and town boundaries dataset (2000–2022) derived from GLC_FCS30D product

**DOI:** 10.1038/s41597-025-06368-9

**Published:** 2025-12-04

**Authors:** Ming Bai, Xiao Zhang, Weitao Ai, Xia Jing, Liangyun Liu

**Affiliations:** 1 College of Geomatics, Xi’an University of Science and Technology, Xi’an, 710054 China; 2 International Research Center of Big Data for Sustainable Development Goals, Beijing, 100094 China; 3 Key Laboratory of Digital Earth Science, Aerospace Information Research Institute, Chinese Academy of Sciences, Beijing, 100094 China; 4 School of Electronic, Electrical and Communication Engineering, University of Chinese Academy of Sciences, Beijing, 100049 China

**Keywords:** Ecology, Environmental sciences

## Abstract

Urban boundaries are essential indicators for understanding spatial structure and dynamic changes in human settlements. Most existing high-resolution urban boundaries datasets performed poorly on distinguishing low-density built-up areas from non-urban spaces and overlooking the role of population distribution in defining urban extents. In this study, we developed a dual-threshold method by integrating impervious surface density and population data to map annual city and town boundaries from GISD30 (global 30 m impervious-surface dynamic dataset). Specifically, we combined kernel density estimation and cellular automata algorithms to generate global urban boundaries, and then differentiated urban settlement types (e.g., cities and towns) based on population thresholds, thereby producing the Global City and Town Boundaries (GCTB) dataset at 30 m resolution for the period 2000–2022. The GCTB dataset achieves strong agreement with the high-resolution urban boundary interpretation dataset—Atlas of Urban Expansion (R² > 0.88). Using OpenStreetMap place tags, the City/Town split reaches precision 0.80 (City) and 0.65 (Town) with overall accuracy 0.75. Therefore, GCTB provides essential spatial information for global urbanization and sustainable-development planning.

## Background & Summary

Urban areas represent the predominant form of human settlements. Their spatial extent and dynamic evolution are closely tied to land use changes^1,2^, ecosystem stability^3–6^, and progress toward sustainable development goals^7^. Rapid urbanization, fueled by global population growth, is leading to the expansion of urban settlements, particularly towns and peri-urban areas. Future urban expansion is projected to extend urban areas toward the edges of 11–33 million hectares of natural habitats, posing serious threats to biodiversity^8,9^. The United Nations estimates that by 2050, 68% of the global population will live in urban areas, up from 55% in 2018^10^. These challenges underscore the urgent need for a globally consistent, high-resolution urban boundaries dataset.

Over the past decades, high-resolution urban boundary mapping has achieved many progress, but these efforts have largely been confined to either small-scale local regions or selected large metropolitan areas^11–15^. This is mainly due to the high computational demands and limited free access to high-resolution satellite imagery. Recently, breakthroughs in cloud computing and the improved accessibility of remote sensing archives have provided great opportunities for global urban boundaries mapping. A series of high-resolution global urban boundaries datasets have been generated, including Global Urban Boundaries^9^, Global Urban-Rural Settlement Dataset^16^, and MODIS Global Urban Product^17^. However, most of these datasets only distinguish between urban and non-urban or urban and rural areas, while overlooking small towns—critical transitional zones between cities and rural areas. Small towns, often situated near ecologically sensitive regions, substantially contribute to habitat fragmentation, infrastructure inequality, and social vulnerability^18–20^. Despite their significance, small towns remain largely underrepresented in global urban datasets. Therefore, a critical gap remains: the absence of globally consistent, high-resolution datasets capable of distinguishing between cities and towns.

Existing methods for delineating global urban boundaries can be broadly classified into three categories. At first, the earliest methods rely on the coarse-resolution optical satellite imagery (e.g., MODIS), which infer urban areas from spectral reflectance and spatial texture patterns^17,21,22^. Although these data are temporally rich and widely available, their coarse spatial resolution (typically 250–1000 m) limits their ability to capture fine-scale urban morphology, particularly in fragmented or peri-urban regions^23^. The second approach utilizes nighttime light (NTL) data, such as DMSP-OLS NTL and NPP-VIIRS NTL, which define urban extents by synthesizing the intensity and location information from NTL^24,25^. However, NTL-based methods are prone to saturation in densely populated areas and overglow in rural or sparsely settled zones, which can lead to significant spatial overgeneralization due to their relatively coarse resolution (≈1 km)^24,26,27^. The third approaches leverages fine-resolution impervious surface product to identify urban extents through contiguous built-up areas^9,28,29^. Operating at finer spatial resolutions (typically 30 m), these methods provide direct representations of physical infrastructure and urban form. Nevertheless, approaches based solely on impervious surfaces tend to incorporate certain towns and large rural settlements into urban boundaries, thereby overestimating the actual extent of urban areas^30^. This highlights the need for a globally consistent dataset that combines fine spatial resolution with the functional differentiation of urban settlement types.

Here, we generate a novel global 30 m annual urban boundaries dataset (GCTB) covering the period from 2000 to 2022, by integrating time-series impervious surface dataset and gridded population data. The dataset distinguishes between cities and towns based on functional population thresholds and provides consistent annual urban boundaries at fine-resolution across the globe. GCTB represents the first global dataset to simultaneously map both cities and towns over a long time series at 30 m. It offers critical dataset for understanding the spatial distribution and temporal evolution of urban settlements worldwide, and contributes to sustainable urban development, land-use management, and global urban governance.

## Methods

### Remote sensing datasets

In this study, we use GISD30 dataset, an annually consistent global impervious surface dataset at 30 m resolution, as the primary input for delineating urban boundaries. The impervious surface data are derived from the GLC_FCS30D land cover product, a globally consistent 30 m dataset generated using dense time-series Landsat imagery and a fine-classification system for 1985–2022^31^. The GISD30 dataset captures the global spatial distribution and temporal dynamics of impervious surfaces from 2000 to 2022, achieving an average classification accuracy of 90.1%^32^. With its high spatial resolution and continuity, GISD30 enables detailed depiction of urban spatial structures, thereby providing a solid foundation for delineating urban boundaries. GISD30 can be freely accessed via Zenodo (https://zenodo.org/records/5220816)^33^.

Population size remains a fundamental criterion for distinguishing urban settlement types, such as cities, towns, and rural areas in both statistical and spatial delineation frameworks^34–36^. To enable population-informed classification, we used the LandScan Global data (LSG: 10.48690/1529167)^37^ with a resolution of approximately 1 km. This dataset offers consistent annual population estimates from 2000 to 2023 by integrating census records, satellite imagery, and ancillary data such as road networks and land cover^38^. Although it was coarser than GISD30, the temporal consistency and global coverage of LSG make it well-suited for identifying low-density settlements like small towns and peri-urban clusters.

To evaluate the accuracy and spatial consistency of our developed GCTB dataset, we performed comparative validation using multiple representative global-scale urban datasets derived from diverse remote sensing and modeling approaches. Table 1 summarizes their key characteristics, including Global Urban Boundaries (GUB; iEarth DataHub: https://data-starcloud.pcl.ac.cn/iearthdata/14)^9^, MODIS Global Urban Product (MGUP; https://www.researchgate.net/publication/339873537_MGUP_annual_global_2001_2018)^17^, Nighttime Light-based Urban Extent (NTL-UE; 10.6084/m9.figshare.16602224.v1)^39^, and Global Urban-Rural Settlement Dataset (GURS; Zenodo: https://zenodo.org/records/11160893)^40^. GUB and GURS offer urban boundaries products at moderate temporal resolution, based on impervious surface mapping and hybrid data integration methods, respectively. The MGUP and NTL-UE offer annual estimations based on coarse MODIS and nighttime light imagery, respectively. The Atlas of Urban Expansion (AUE; https://atlasofurbanexpansion.org/data)^11^ is manually delineated based on high-resolution satellite imagery and official statistical data—offering relatively high spatial accuracy—it focuses only on approximately 200 large cities worldwide. Therefore, they are used as the reference datasets for quantitative validation at the regional or metropolitan scale.

**Table 1:** Overview of the datasets involved in the comparison and verification (including this study).

Dataset	Full Name	Temporal Coverage	Spatial Resolution	Data Source	Methodology	Accuracy
GUB	Global Urban Boundaries	2000–2018, every 5 years	30 m	GAIA (Global Artificial Impervious Area)	Morphological clustering of impervious surface data	—
MGUP	MODIS Global Urban Product	2001– 2018, annual	250 m	MODIS optical imagery	Automated sampling + RF classification	F1 ≈ 0.88 (global)
NTL-UE	Nighttime Light-based Urban Extent	1992–2020, annual	1 km (DMSP-OLS), 500 m (VIIRS); harmonized to 1 km	DMSP-OLS and VIIRS	Light intensity thresholding	—
GURS	Global Urban-Rural Settlement Dataset	2000–2020, every 5 years	100 m	GHS-BUILT-S R2023A + VIIRS + functional zoning	Hybrid fusion of impervious surface, NTL, and zoning data	Overall accuracy ≈ 91.2%, K ≈ 0.85 (global)
AUE	Atlas of Urban Expansion	1990–2014 (selected years)	≈30 m (variable)	Official planning maps, satellite imagery	Expert interpretation and manual delineation	Overall accuracy ≈ 87–89% (multi-city; protocols vary)
GCTB (This study)	Global City & Town Boundaries	2000–2022, annual	30 m	GISD30 impervious surface; LandScan population	Dual-threshold of impervious & population and patch-level aggregation	Overall accuracy ≈ 75%; (OSM validation)

### Methodological framework

**Fig. 1:**
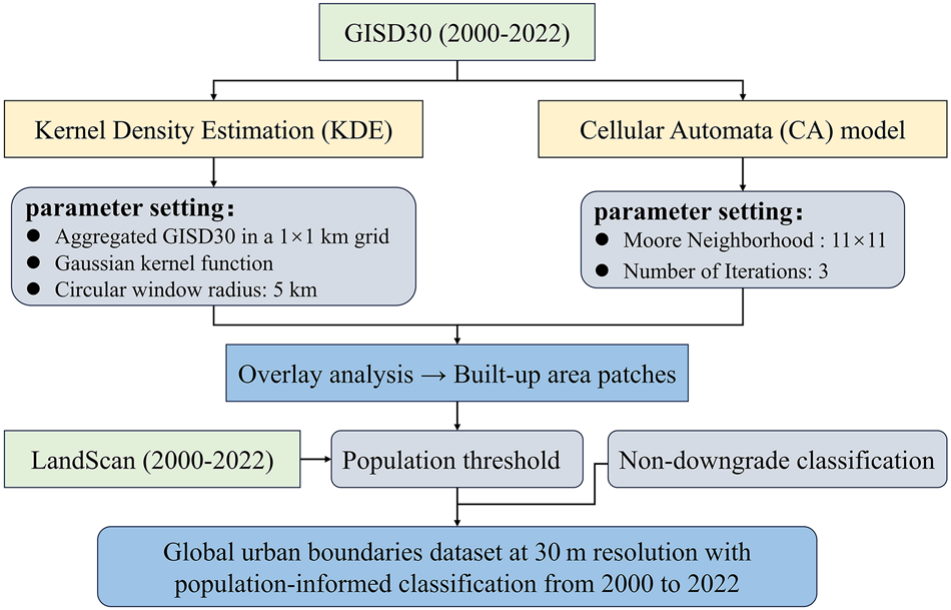
Workflow for generating the Global City and Town Boundaries (GCTB) dataset.

Figure 1 illustrates the overall methodological framework of the proposed method. It mainly contains three key components: identifying these high-density urban cores from GISD30 products using aggregation and kernel density estimation smoothing^41^, refining boundary continuity using cellular automata models^42^, and importing the population information to further split the cities and towns.

### Built-up area patches identification

To ensure the completeness and morphological integrity of built-up area delineation, we adopted a two-stage approach based on the framework proposed by Li *et al*.^9^. This framework combines Kernel Density Estimation (KDE) with a rule-based Cellular Automata (CA) model to improve the spatial aggregation and connectivity of urban forms. This approach addresses two key limitations of conventional pixel-based impervious surface classification: (1) poor spatial aggregation in low-density areas, and (2) fragmented or discontinuous boundaries at the urban fringe.

Specifically, urban expansion typically occurs in clusters with high impervious surface density, however, raw 30 m resolution data (e.g., GISD30) often contain high-frequency noise and isolated patches, especially in peri-urban and low-density areas. To reduce local speckle and ensure cross-national consistency, we upscale 30 m impervious pixels to the fraction of impervious surfaces at 1 km, and the choice of the upscaling scale (1 km) corresponded to works of United Nations(UN)-Organisation for Economic Co-operation and Development(OECD) Degree of Urbanization, which believed that urban population statistics derived from a 1 km² spatial grid effectively ensure the comparability of statistical data across different countries^43^. Meanwhile, the upscaling scale of 1 km was demonstrated to efficiently suppress pixel-level noise problem and retain sufficient spatial detail for subsequent boundary identification^44^. Next, we applied a Gaussian kernel density estimation (KDE) to the aggregated ISA layer with a 5 km search radius (bandwidth). This choice aligns with the 5-km moving window used by the USGS Terrestrial Development Index and was empirically selected after comparing radius from 1 km to 10 km^45,46^. This method enhances spatial clustering and suppresses isolated noise by generating a continuous surface of impervious surface intensity, as shown in Eq. (1):

1 $$f(x,y)=\frac{1}{n{h}^{2}}\mathop{\sum }\limits_{i=1}^{n}K\left(\frac{x-{x}_{i}}{h},\frac{y-{y}_{i}}{h}\right)$$

where *K* is the Gaussian kernel function, which defines the weight contribution of each ISA pixel within the smoothing window based on spatial proximity The parameter ℎ represents the bandwidth, which corresponds to the radius of the smoothing window, while *n* denotes the total number of impervious surface (ISA) pixels contained within the window and involved in the density estimation. (${x}_{i}$, ${y}_{i}$) are the spatial coordinates of the sample pixels with associated ISA values. KDE has been widely used in urban morphology studies due to its ability to reveal urban cores through spatial smoothing^41,47^. We thresholded the KDE-smoothed imperviousness surface at f(x,y) ≥ 0.20 to extract urban cores, f(x,y) denotes the kernel-weighted impervious fraction within a 5-km Gaussian window. The 20% threshold coincides with the National Land Cover Database boundary for separating Open Space and Developed, which was widely used to preserve contiguous urban structure while limiting fringe scatter^48,49^. In line with established practice, this threshold retains high-density, functionally continuous urban structure while minimizing inclusion of scattered peri-urban development.

Urban cores alone may underestimate actual urban extents, particularly in peripheral regions where fragmented but functionally connected built-up patches exist. To address this issue, we applied a non-predictive CA model at 30 m resolution with a Moore neighborhood window size of 11 × 11 pixels to refine boundaries by enhancing spatial connectivity. It should be noted that the choice of window size of 11 pixels came from the work of Kocabas & Dragićević (2006)^50^. We iteratively ran the urban CA model three times, yielding an expansion distance of ≈1 km (≈33 pixels). These settings are consistent with evidence that neighborhood size and type materially affect CA outcomes, with larger windows reducing patch fragmentation and shortening total edge length; our 11 × 11 choice falls squarely within the radius 2–10 configurations systematically evaluated by Kocabas & Dragićević (2006)^50^. This approach allows controlled boundary expansion (≈1 km), connecting spatially close but disjoint patches, thus producing compact and coherent urban boundaries that better reflect the morphological reality. As shown in Fig. 1, these two modules—KDE smoothing and CA refinement—jointly improve the morphological integrity of urban boundaries, producing compact and realistic delineations across a variety of urban forms.

### Boundaries refinement and post processing

It is noteworthy that the urban patches derived from the cellular automata model still suffered the sharp corners and noisy outlines due to grid effects, thus, a series of spatial refinement steps are proposed to enhance morphological integrity, and ensure the vector usability of the final boundaries. This post-processing pipeline addresses common issues in urban boundary delineation, including jagged edges, internal voids, and artificial disconnections.

Specifically, to harmonize the morphology step with the 30 m grid and the 1 km smoothing scale, we applied morphological closing (binary dilation followed by erosion) using a square structuring element, following the prior works of UGB (urban growth boundaries) with the dilation/erosion slide window of 7 × 7^51^. The 7 × 7 kernel is large enough to bridge narrow gaps and eliminate staircase artifacts introduced by the raster grid, yet compact enough to preserve neighborhood-scale form. This operation regularizes patch perimeters and suppresses spurious crenellations with only marginal effects on area, thereby improving the geometric integrity and vector usability of the resulting boundaries^51,52^.

Urban patches often contain internal holes caused by water bodies, green spaces, or large open areas with low impervious surface coverage. Although these regions may not exhibit strong impervious surface signals, they are functionally part of the urban landscape. To address this issue, we applied a topology-based hole-filling procedure that merges completely enclosed non-urban areas into the surrounding urban polygon if they are fully surrounded by urban pixels^9^. This method ensures the morphological continuity and topological completeness of each urban unit, thereby enhancing the consistency and usability of the final vector dataset for spatial analyses such as area computation and zoning.

In some cases, small peripheral patches remain disconnected from the urban core due to sparse impervious surface coverage, even though they are functionally integrated (e.g., walkable distance, shared services). To reduce fragmentation and enhance functional continuity, we merged built-up patches located within 1 km of the identified urban cores. This distance threshold is consistent with urban planning standards that define 1 km as a typical walkability range and has been widely adopted in previous studies^28^. This spatial merging strategy ensures that compact clusters and peripheral fragments are treated as unified urban entities, resulting in cleaner and more realistic urban extents.

### Urban classification

A framework for distinguishing cities and towns based on population using the United Nations Degree of Urbanization: Classifying urban patches into cities (≥50,000) and towns (5,000 to 50,000)^34^. We adopted a population-based classification framework using annual gridded population estimates from the LandScan Global dataset (2000–2022). Although LSG has a relatively coarse resolution (~1 km), it provides temporally consistent and globally comparable population estimates derived from census data and ancillary inputs such as land cover, transportation networks, and built-up areas. Each urban patch was overlaid with the annual LSG population grid to estimate the total population within its spatial footprint. This typology follows international statistical standards and enables consistent classification across countries, independent of local administrative designations. It also aligns with recent efforts by the European Union (EU)-OECD and UN to harmonize urban definitions globally^36^. It is important to note that we adopted only the urban population totals from the definitions and gave up the criterion of population-density because some intra-urban open spaces are filled to maintain morphological continuity, i.e., imposing a density threshold alongside population counts would systematically understate urban in areas with locally low density.

Annual gridded population estimates may exhibit minor fluctuations due to modeling uncertainty or temporal variability (e.g., census lags). To avoid spurious year-to-year classification changes, we implemented a non-downgrade rule to maintain label stability: once an urban patch is classified as a city in the base year (2000), its status is retained in all subsequent years, regardless of temporary population decline. This rule enforces temporal consistency and mitigates artificial “downgrades” that may not reflect actual functional changes on the ground. Although this approach may introduce inertia in regions experiencing long-term population decline, it ensures coherent temporal trajectories and minimizes classification noise.

To verify the accuracy of our City and Town labels, we used OpenStreetMap (OSM) as an external crowdsourced reference (© OpenStreetMap contributors; data available at https://www.openstreetmap.org)^53^. Specifically, we extracted features tagged place = city, place = suburb, and place = town (case-insensitive), following OSM tagging definitions (see Key: place and related tag pages). Because suburbs are functionally attached to metropolitan cores, we treated place = suburb as City in the main analysis.

## Data Records

The developed global annual urban boundary dataset at 30 m resolution (2000–2022) is openly accessible via the Zenodo platform (https://zenodo.org/records/16418717)^54^. It documents the spatial extents of cities and towns over 23 years, incorporating global coverage and population-informed functional classification. Each annual release includes two vector layers: Cities_Year (city boundaries) and Towns_Year (town boundaries). Here, Year denotes the specific year of observation (e.g., Cities_2000, Towns_2000). All data layers are projected in the WGS 84 geographic coordinate system (EPSG:4326) and delivered in vector polygon format.

**Fig. 2:**
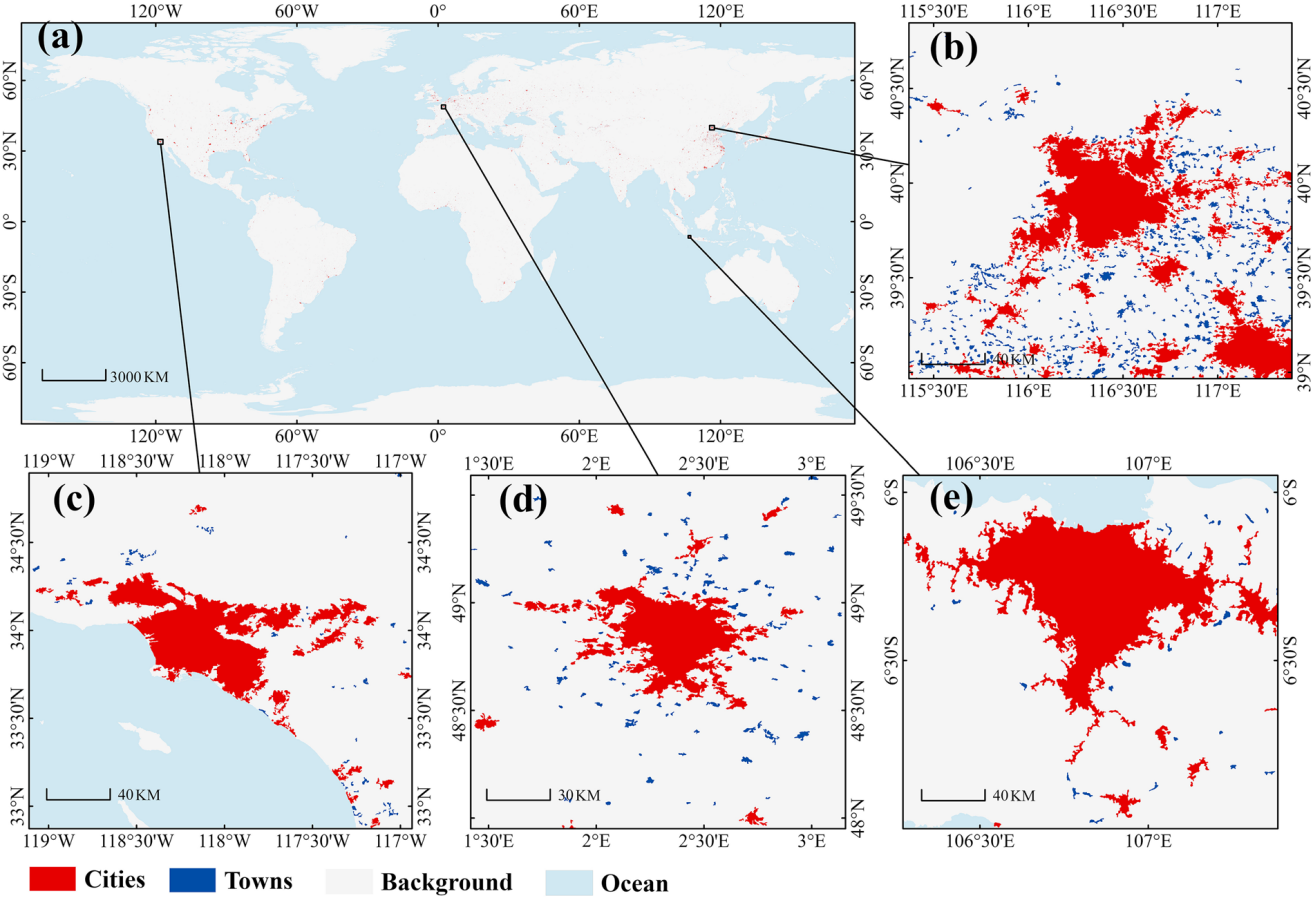
Spatial distribution of global cities and towns in 2022. (**a**) Global map showing cities (red) and towns (blue) at 30 m resolution. (**b**) Beijing–Tianjin–Hebei region, China; (**c**) Los Angeles metropolitan area, USA; (**d**) Paris region, France; (**e**) Jakarta metropolitan area, Indonesia.

Figure 2 illustrates the global urban boundary distribution derived from the GCTB dataset for the year 2022. At the global scale (Fig. 2a), the dataset captures extensive and scattered urban settlements across all inhabited continents, with clear differentiation between cities and towns. To further demonstrate the dataset’s delineation accuracy and spatial detail, four representative regions are shown at higher resolution: Beijing (Fig. 2b), Los Angeles (Fig. 2c), Paris (Fig. 2d), and Jakarta (Fig. 2e). These enlargements highlight the ability of the dataset to distinguish compact urban cores from surrounding lower-density settlements, and to represent fragmented or irregular urban forms across diverse geographic contexts.

## Technical Validation

To assess the spatial accuracy and reliability of the Global Urban Boundaries (GCTB) dataset, we conducted a comprehensive validation using multiple independent sources, including satellite-derived and authoritative reference datasets. Validation was performed using three approaches: (1) cross-comparison with existing global urban datasets, (2) overlaying historical high-resolution imagery, and (3) regression analysis against the Atlas of Urban Expansion (AUE) dataset.

### The overview of the GCTB using historical imagery

**Fig. 3:**
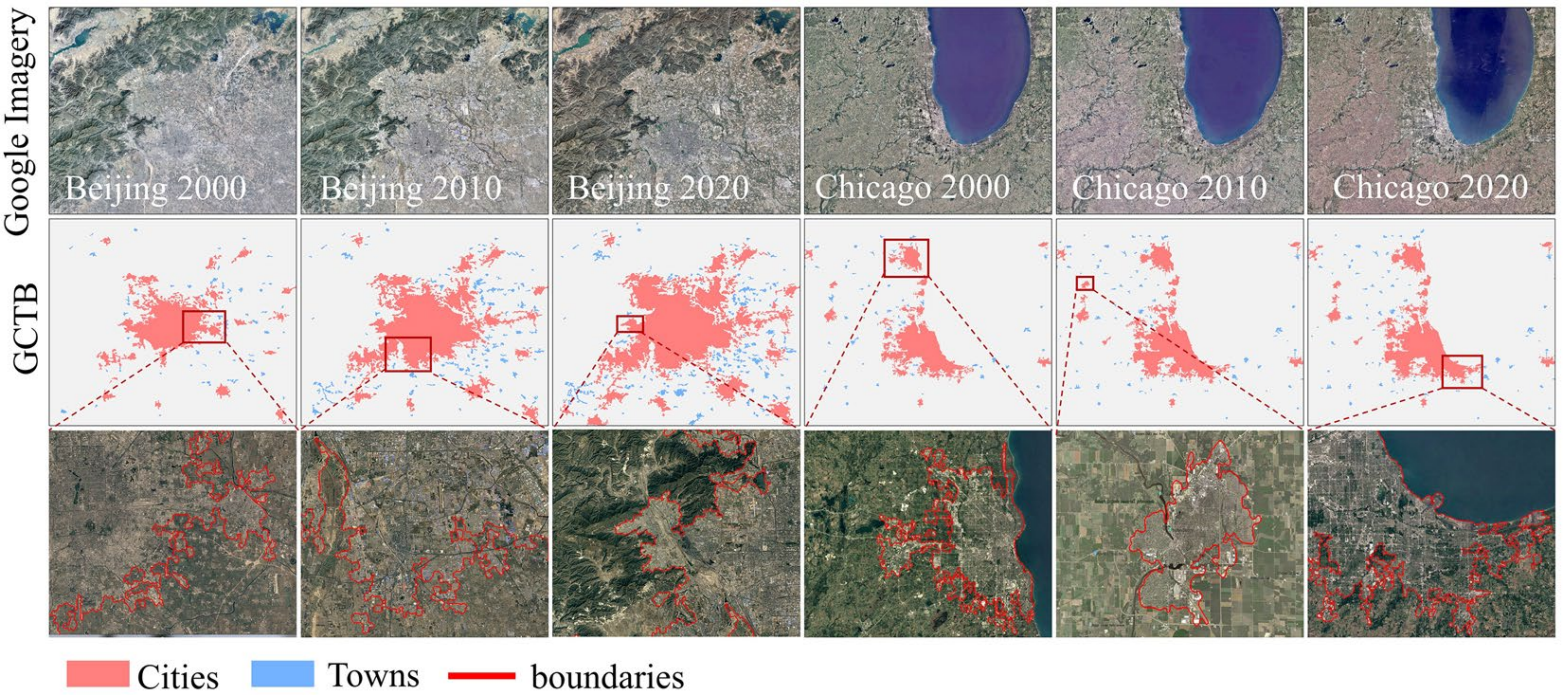
Urban boundary overlays for Beijing and Chicago in 2000, 2010, and 2020: GCTB City (red) and Town (blue) polygons draped on Google satellite imagery; red outlines denote derived boundaries. Insets magnify boxed areas to illustrate fringe morphology and boundary adherence.

To understand the performance developed GCTB dataset, we selected two representative cities: Beijing in China and Chicago in the United States. We conducted a comparative analysis using historical high-resolution imagery from Google Earth across three benchmark years spanning 2000 to 2022: 2000, 2010, and 2020 (Fig. 3).

The results indicate that GCTB accurately captures the distinct spatio-temporal evolution patterns of both cities. Beijing (Rapid Expansion and Transformation): Over the three observation years (2000–2020), GCTB clearly delineates the significant outward expansion of Beijing’s urban boundary, closely matching the actual spatial extent of built-up areas visible in satellite imagery. Crucially, GCTB successfully tracks both the explosive growth of peri-urban zones and the infill development within previously underdeveloped interior areas. At the town-level scale, the boundaries of surrounding towns show a clear pattern: they either remain relatively stable in the earlier years before being gradually incorporated into the expanding urban core in later years (e.g., 2010–2020), or their boundaries shift dynamically, directly reflecting their transformation from rural to urban land uses over this period. Chicago (Stable Growth & Persistent Structure): In contrast, across the same time period (2000–2020), Chicago exhibited more moderate and structurally consistent growth. GCTB accurately maintains the delineation of the established core city boundaries with minimal major shifts. More importantly, it consistently identifies and delineates persistent suburban municipalities and township regions throughout all three years. While internal infill development occurs, the overall pattern captured by GCTB is one of stability in the broader metropolitan boundary and the persistent existence of distinct suburban towns.

### The consistency analysis between GCTB and four comparative datasets

To systematically assess and temporal consistency of the GCTB dataset in delineating urban boundaries, we applied two complementary cross-validation approaches and compared the results against four widely used global urban boundary datasets: GUB, GURS, MGUP, and NTL-UE.

**Fig. 4:**
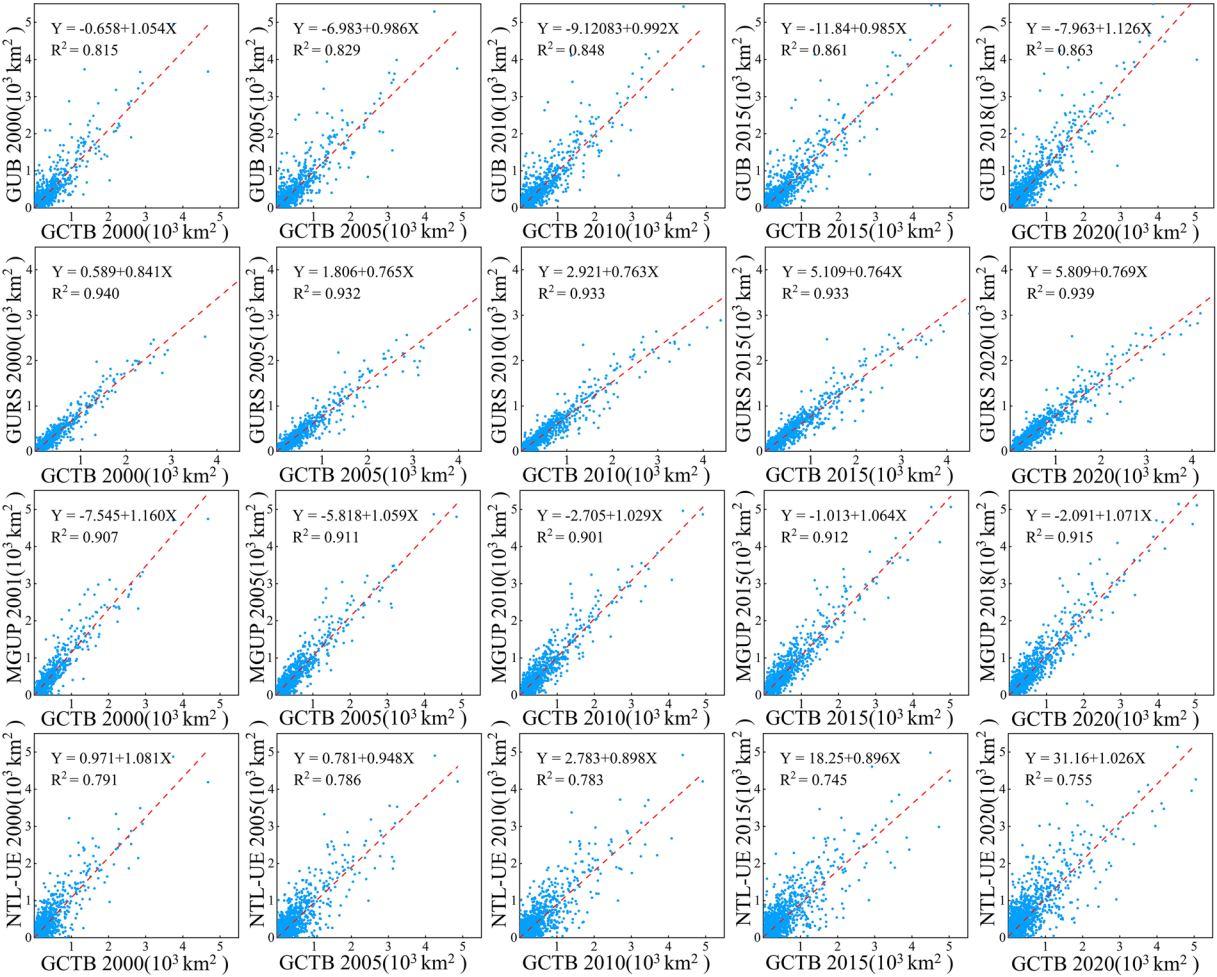
Scatterplot comparisons of urban area between the GCTB dataset and four global urban boundary datasets (GUB, GURS, MGUP, and NTL-UE) at five benchmark years (2000, 2005, 2010, 2015, and 2020). Urban areas were aggregated within global 1° × 1° grid cells. The horizontal axis represents the GCTB-derived urban area size, while the vertical axis shows the urban area size from the reference dataset.

We calculated the total urban area within 1° × 1° grid cells for five benchmark years (2000, 2005, 2010, 2015, and 2020), and created scatterplots to compare GCTB with the four reference datasets (Fig. 4). The results show that GCTB shows the highest consistency with GURS, with R² values consistently exceeding 0.93 and regression slopes close to 1, indicating strong spatial agreement in urban boundary delineation. This high level of agreement is partly attributed to GURS’s methodological distinction between urban and rural areas, which effectively reduces the misclassification of low-density or non-urban spaces as urban extents. GCTB also exhibits relatively good consistency with MGUP and GUB, with R² values generally ranging from 0.85 to 0.91, though overall agreement is slightly lower than with GURS. Both MGUP and GUB show a tendency to overestimate urban boundaries by including rural settlements or non-functional built-up areas, leading to boundary expansion and potential overgeneralization at urban fringes. In contrast, NTL-UE displays the lowest consistency with GCTB, with R² values typically below 0.80 and regression slopes significantly less than 1, indicating systematic underestimation of urban extents. This bias primarily results from the coarse 1 km spatial resolution of NTL-UE, which limits its ability to detect scattered built-up areas, as well as potential signal saturation in high-density urban cores when using nighttime light data.

**Fig. 5:**
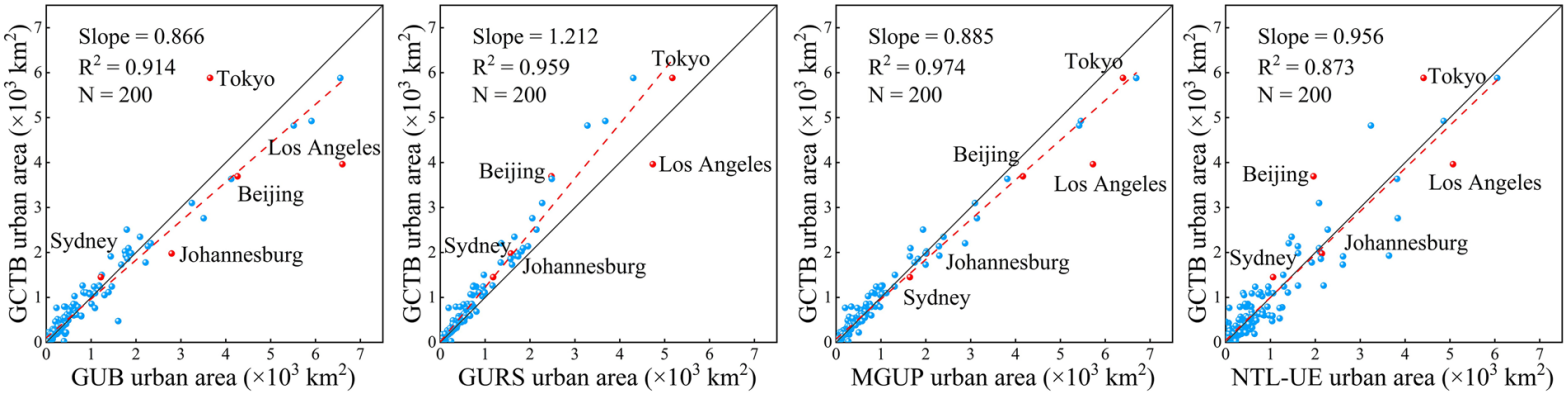
City-wise consistency between GCTB and four comparative datasets in 2015 (N = 200). The scatter plot compares GCTB city areas (y-axis) with GUB, GURS, MGUP, and NTL-UE city areas (x-axis). The gray line denotes the 1:1 reference. Panel annotations report the regression slope and R^2^.

To complement the grid-based assessment, we created city-scale consistency scatter plots for 200 cities from the AUE database (Fig. 5). The results show that GCTB has a strong fit with GUB, GURS, and MGUP (R^2^ > 0.90), while it suffers the lowest consistency with NTL-UE mainly due to its coarse spatial resolution and saturation problem of nighttime-light. To illustrate these differences, we selected five representative cities: In Los Angeles, GCTB is smaller than MGUP and NTL-UE, slightly larger than GUB, and comparable to GURS. In Tokyo, GCTB is larger than GUB and GURS, similar to MGUP, but smaller than NTL-UE. In Sydney and Johannesburg, GCTB is smaller than all four reference datasets, with broadly consistent differences across years. Overall, GCTB is smaller than GUB/MGUP/NTL-UE because its boundary classification distinguishes City from Town and excludes small, fragmented built-up patches. Conversely, GCTB is larger than GURS because GURS retains internal open spaces at the 100-m pixel scale, whereas GCTB fills and consolidates these internal voids.

### Comparison with global urban time-series datasets

To intuitively understand the spatial accuracy, temporal consistency, and morphological integrity of the GCTB dataset, we performed a multi-temporal comparison with four widely used global urban datasets (GUB, MGUP, GURS, and NTL-UE) and two high-resolution reference layers (GISD30 and AUE). Comparisons were conducted for five benchmark years: 2000, 2005, 2010, 2015, and 2020. Six representative cities—Milan (Italy), Philadelphia (USA), Sydney (Australia), Buenos Aires (Argentina), Johannesburg (South Africa), and Wuhan (China)—were selected to capture diverse regional, morphological, and developmental characteristics across continents.

**Fig. 6:**
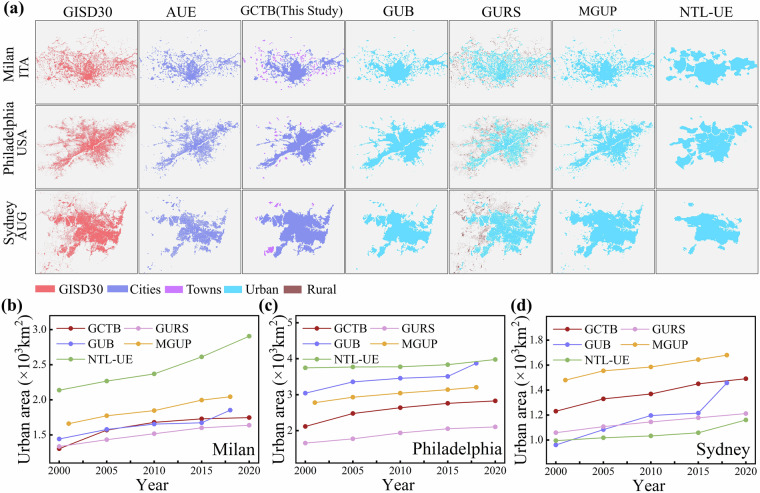
The spatial comparisons between GCTB and five other urban boundary datasets in 2015 (**a**), and the time-series urban area statistics during 2000–2020 in Milan, Philadelphia and Sydney (**b**–**d**).

Figure 6 presents the comparison of the 2015 urban boundaries and temporal area changes for three representative cities in developed countries: Milan, Philadelphia and Sydney. For Milan (Fig. 6a,b), all datasets delineate the core built-up area well, but they diverge at the periphery. NTL-UE shows the greatest outward expansion, reflecting ~1-km scale effects and nighttime-light diffusion. MGUP tends to merge with low-density residential belts and transition zones. GURS preserves internal open spaces, resulting in fragmented and smaller boundaries. GUB provides precise delineation but shows localized overexpansion. Constrained jointly by morphology and population at 30-m resolution, GCTB maintains continuity while limiting excessive expansion and shows stable, moderate growth over time. Philadelphia (Fig. 6a,c) shows a similar pattern. NTL-UE remains the largest and fastest-growing estimate, and GUB and MGUP are generally larger than GCTB. GURS is the smallest with the slowest growth. GCTB aligns most closely with the high-resolution references (GISD30/AUE) and shows no evident spillover. Sydney (Fig. 6a,d) shows the same pattern of core consistency and peripheral divergence. After 2015, GUB grew rapidly, periodically widening its gap with GCTB, whereas GCTB maintained restrained expansion with gradual convergence over time. NTL-UE remained the largest, followed by MGUP; GURS was the smallest. Across the three cities, NTL-UE’s expansion is mainly driven by nighttime-light diffusion and scale effects; MGUP tends to incorporate low-density peripheries; GUB shows localized overexpansion in specific areas; and GURS remains smaller because it retains pixel-level voids. In contrast, GCTB most closely matches the high-resolution references in both spatial morphology and temporal trends. It suppresses the spurious inclusion of peripheral low-density or non-functional built-up areas, yielding a more constrained and temporally stable sequence of urban boundaries.

**Fig. 7:**
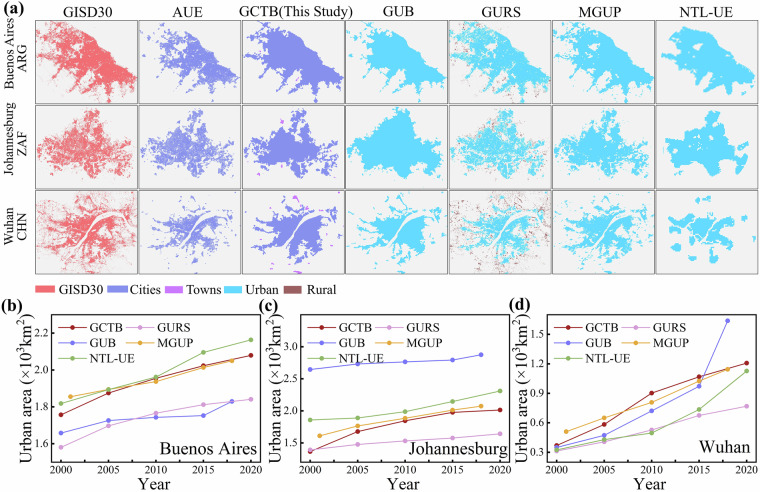
The spatial comparisons between GCTB and five other urban boundary datasets in 2015 (**a**), and the time-series urban area statistics during 2000–2020 in Buenos Aires, Johannesburg and Wuhan (**b**–**d**).

As shown in Fig. 7, a comparative analysis of the 2015 urban boundaries and a temporal comparison of the total areas were conducted for three cities exhibiting fragmented and dispersed forms: Buenos Aires, Johannesburg and Wuhan. Consistent with the above, all datasets identify the urban core in the spatial overlays (Fig. 7a). In Buenos Aires (Fig. 7a,b), the datasets agree on the core but diverge at the periphery. NTL-UE yields the largest extents; MGUP and GUB tend to absorb low-density fringes; and GURS remains smaller because it retains pixel-level voids. GCTB aligns most closely with AUE and shows steady growth. Johannesburg (Fig. 7a,c) shows a stronger contrast in expansion patterns. GUB exhibits the most pronounced contiguous outward expansion, followed by NTL-UE; MGUP is intermediate, and GURS is the most conservative. GCTB maintains continuity and converges steadily toward the references. Wuhan (Fig. 7a,d), shaped by river networks and corridor structures, shows greater cross-dataset divergence in riparian zones. GUB and MGUP incorporate more area on both sides of the corridors; NTL-UE expands in blocky patterns; and GURS retains large internal voids. By contrast, GCTB and AUE exhibit the most consistent geometry. Across cities, the patterns are consistent: NTL-UE’s larger size mainly reflects scale effects and nighttime-light diffusion; GUB and MGUP expand readily into transition zones; GURS remains smaller because it retains voids; and, under joint constraints of 30-m morphology and population, GCTB shows continuous yet restrained temporal expansion and aligns most closely with the high-resolution references.

### Validation of urban extents using the Atlas of Urban Expansion (AUE)

**Fig. 8:**
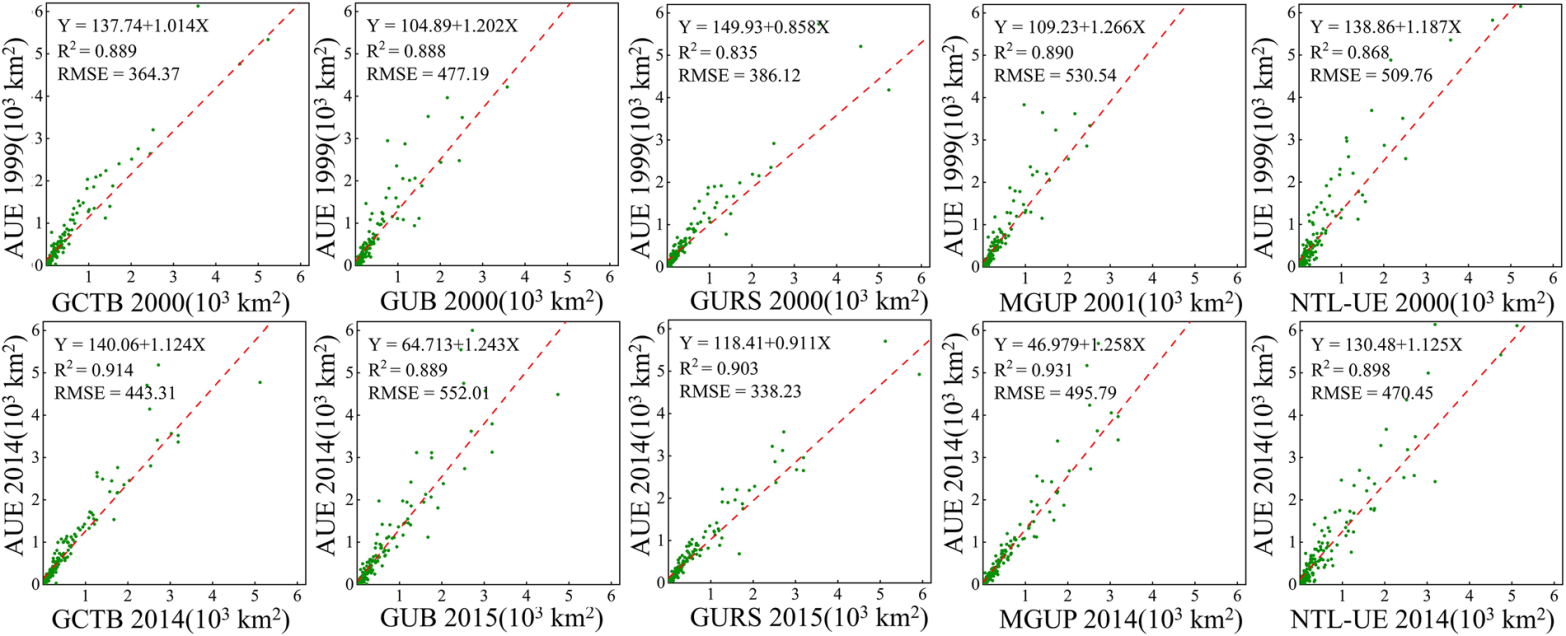
Scatterplot comparisons of urban area estimate from GCTB and four global urban boundary datasets (GUB, GURS, MGUP, and NTL-UE) against the Atlas of Urban Expansion (AUE) for the years 1999 and 2014. Each panel presents the linear regression between AUE and a given dataset, along with the corresponding regression equation, R^2^ value, and Root Mean Square Error (RMSE).

To further understand the accuracy of GCTB in delineating urban boundaries, we conducted a comparative analysis using the widely recognized Atlas of Urban Expansion (AUE) dataset for two benchmark years, 1999 and 2014. As shown in Fig. 8, scatterplots assess the agreement between AUE and five global urban boundary datasets: GCTB, GUB, GURS, MGUP, and NTL-UE. GCTB demonstrated the strongest agreement and highest accuracy, achieving the highest R² values (0.889 in 1999; 0.914 in 2014) with regression slopes close to 1 (1.014 and 1.124, respectively), and the lowest Root Mean Square Error (RMSE) in both years. This exceptional alignment with AUE, based on high-resolution imagery and expert interpretation, underscores GCTB’s credibility and delineation precision. GURS also performs well but varies by year—R^2^ = 0.835 (2000) and 0.903 (2015) in our panels and among the lowest RMSE in 2015 (≈358 km²). In contrast, GUB, MGUP, and NTL-UE all exhibited notable deviations. For example, both GUB and MGUP showed regression slopes greater than 1.2 in 2014, indicating systematic overestimation of urban areas, which was also reflected in their high RMSE values (particularly for MGUP and GUB). NTL-UE likewise showed slopes greater than 1 (1.187 in 1999 and 1.125 in 2014), suggesting a similar overestimation tendency. While GUB and MGUP tend to overestimate small towns and large rural settlements within urban boundaries, while the overestimation of NTL-UE is primarily caused by the light bloom effect under its coarse spatial resolution, which leads to outward boundary expansion. In summary, GCTB demonstrated the highest spatial consistency and the lowest estimation error relative to AUE, reinforcing its reliability as a globally consistent and accurate dataset for urban boundary mapping.

### OSM-based validation of City vs Town classification

To independently evaluate GCTB’s city–town classification, we built a global place-tag reference using OpenStreetMap (OSM) features. We merged place = city and place = suburb into City, classified place = town as Town, and grouped remaining tags (e.g., district, locality, village, island) into Other. For 2015, 2020, and 2022, we matched OSM points to GCTB city/town polygons using a point-in-polygon test in a unified equal-area projection. We then computed precision, recall, F1, and overall accuracy for the city and town subsets. The evaluation set contained 84,046 points (2015), 123,177 (2020), and 138,177 (2022), for a total of 345,400 points. Results are summarized in Table 2. The City class achieved consistently high agreement (F1 > 0.7), whereas the Town class showed moderate agreement (F1 ≈ 0.6–0.7); overall accuracy was ≈0.75. Urban metrics remained stable, whereas town metrics showed lower recall, reflecting regional tagging practices and scale heterogeneity. Because OSM place tags are name-based rather than official population designations, this assessment provides a conservative, independent validation that supports the feasibility and practical utility of GCTB’s city–town distinction at the global scale.

**Table 2:** OSM-based evaluation of GCTB class labels (City vs Town) for 2015, 2020, and 2022.

Year	Class	Precision	Recall	F1	Support	Overall Accuracy	Samples
2015	City	0.779	0.86	0.817	51,848	0.763	84,046
	Town	0.729	0.607	0.662	32,198		
2020	City	0.784	0.834	0.808	78,791	0.747	123,177
	Town	0.668	0.593	0.628	44,386		
2022	City	0.78	0.823	0.801	87,343	0.742	138,177
	Town	0.664	0.602	0.632	50,834		

## Usage Notes

The high-resolution global annual urban boundaries dataset developed in this study spans the period from 2000 to 2022 and captures the spatiotemporal evolution of cities and towns. It provides essential spatial support for a wide range of applications, including urban expansion monitoring, sustainable development assessment, and land-use analysis. To ensure the accuracy and consistency of boundary delineation, we integrated temporally stable impervious surface data, spatially coherent population data, and a population-informed urban classification scheme. Accuracy assessments and cross-comparisons with existing global datasets confirm that the dataset effectively captures the spatial structure and temporal dynamics of urban settlements worldwide.

Nonetheless, several considerations remain, along with potential remedies. Because GCTB’s delineation relies on GISD30 and LSG, residual upstream errors (e.g., omissions in complex terrain or under persistent cloud cover) may propagate and reduce the completeness of the impervious-surface–based outlines^9,55^. Since LSG represents a rasterized and ambient 24-hour population surface, that is, it can underestimate these dense and clustered towns and overestimate peri-urban areas, and it does not explicitly capture commuting flows or residence–workplace separation. These effects are most consequential for patches near the City/Town size thresholds^56–58^. In GCTB, LSG is used only once—after 30-m morphological boundaries are finalized—to aggregate patch-level population and assign City/Town labels using population thresholds (50,000 and 5,000). Consequently, boundary geometry is unaffected by population, and evaluating whole patches (rather than pixels) further reduces bias outside the threshold bands. Looking ahead, we will evaluate: (i) fusion of standardized 1-km population grids (GHS-POP, WorldPop, LSG) with region-specific weighting; (ii) an optional density-constrained labeling in which patches falling below regionally calibrated, DoU-consistent density proxies are flagged. We will also report per-patch diagnostics—population, proxy density, and ±10% “borderline” flags—to support this evaluation^57–61^. Despite multi-source fusion and a cellular-automaton–based step to enhance connectivity, highly heterogeneous agglomerations—especially rapidly urbanizing peri-urban belts—may still appear fragmented or over-smoothed. These cases will require targeted refinement strategies in future iterations^62^.

Future work will focus on enhancing the robustness and flexibility of the classification framework by: Integrating additional functional indicators (e.g., nighttime lights, traffic flow, and POI data) to enhance the differentiation of settlement types^63–65^; Incorporating multi-source satellite imagery (e.g., Sentinel-1 and Sentinel-2) to improve the temporal reliability and spatial precision of boundary detection^66^; developing regionally adaptive models to better account for spatial heterogeneity and extend the applicability of the method across diverse geographic contexts.

## Data Availability

The GCTB dataset is openly available on Zenodo at 10.5281/zenodo.16418717^54^.

## Code availability

The code used to generate the GCTB dataset is publicly available at https://github.com/baiming226120/GCTB_Code/blob/main/GCTB_Code. The initial extraction of high-density urban cores and preliminary urban boundaries was implemented on the Google Earth Engine (GEE) platform using JavaScript APIs. Subsequent post-processing procedures, including morphological filtering, void filling, and functional classification, were conducted in ArcGIS Pro using Python-based geoprocessing workflows. The repository provides detailed scripts and documentation to reproduce the entire boundary generation pipeline.
